# Midsagittal Midbrain Area and Midbrain-to-Pons-Ratio Cannot Distinguish Overlap Syndromes Between Amyotrophic Lateral Sclerosis and Progressive Supranuclear Palsy

**DOI:** 10.1007/s00062-025-01564-x

**Published:** 2025-09-12

**Authors:** Daniel Cantré, Jochem König, Caroline Makowsky, Martin Dyrba, Johannes Prudlo

**Affiliations:** 1 Institute of Diagnostic and Interventional Radiology, Paediatric Radiology and Neuroradiology, Rostock University Medical Centre, Ernst-Heydemann-Str. 6, 18057 Rostock, Germany; 2 Institute of Medical Biostatistics, Epidemiology and Informatics, University Medical Centre, Johannes Gutenberg University Mainz, Mainz, Germany; 3 Department of Neurology, Rostock University Medical Centre, Rostock, Germany; 4 German Centre for Neurodegenerative Diseases (DZNE), Rostock, Germany

**Keywords:** Amyotrophic lateral sclerosis, Progressive supranuclear palsy, Frontotemporal dementia, Morphometric MRI

## Abstract

**Purpose:**

When amyotrophic lateral sclerosis (ALS), a TDP-43 proteinopathy, and progressive supranuclear palsy (PSP), a tauopathy, are associated with frontotemporal dementia (ALS-FTD or PSP-FTD), clinical differentiation can be challenging. There are no established imaging biomarkers to differentiate ALS-FTD from PSP-FTD.

**Methods:**

We evaluated the midsagittal midbrain area (MBA) and the midbrain-to-pons-(MB/P)-ratios in T1 MPRAGE MRI of 36 PSP cases (*n* = 14 PSP-FTD), 77 ALS cases (*n* = 10 ALS-FTD), and 72 healthy controls (HC).

**Results:**

In ALS, both parameters were indistinguishable from HC. Patients with ALS-FTD had low MBA-values and MB/P-ratios not significantly different from cases of PSP. While ROC-analyses provided an excellent diagnostic accuracy of both parameters for differentiating PSP from HC (AUC_MBA_ = 0.974) as well as PSP from ALS (AUC_MBA_ = 0.982), midbrain morphometry provided poor diagnostic accuracy for distinguishing ALS-FTD from PSP-FTD (AUC_MBA_ = 0,614).

**Conclusion:**

The MBA and the MB/P-ratio are morphometric parameters that have proven reliable in atypical Parkinsonian syndromes. Both can distinguish between PSP and ALS in their typical clinical forms. However, they cannot differentiate between PSP-FTD and ALS-FTD.

## Introduction

Amyotrophic lateral sclerosis (ALS) is a TDP-43 proteinopathy, whereas progressive supranuclear palsy (PSP) is a tauopathy. Both diseases can be associated with frontotemporal dementia (FTD) that is based on either TDP-43 or Tau pathology. The clinical differentiation of PSP and ALS can be extremely challenging when upper motor neuron signs are dominant in combination with FTD (PSP-FTD & ALS-FTD). This challenge is due to the fact that PSP (defined as 4 repeat tauopathy type PSP) can be detected as primary lateral sclerosis (PLS) [1, 2], and ALS (defined as TDP-43 proteinopathy) can be associated with extrapyramidal symptoms [3], especially with its upper motor neuron variants, and even with vertical gaze palsy in exceptions [4]. Therefore, the reliable differentiation of PSP-FTD and ALS-FTD would be pivotal for providing therapies in such overlap syndromes. Two neuroimaging markers, the midsagittal midbrain area (MBA) and midbrain-to-pons-ratio (MB/P-ratio), have already been shown to differentiate PSP patients from healthy controls and from those with other parkinsonian syndromes [5]. The aim of this study was to investigate whether the MBA and the MB/P-ratio can also differentiate both histopathological entities.

## Material and Methods

### Participants

The study was performed according to the Declaration of Helsinki in its present form. Ethical approval was given by the respective review boards. Written and oral informed consent was obtained from all participants prior to inclusion into the respective DESCRIBE registry-based studies of the German Centre for Neurodegenerative Diseases (DZNE) [6, 7]. Inclusion criteria were patients with either PSP as diagnosed according to MDS-PSP-criteria [8] for the PSP-cohort and patients with ALS according to the revised El Escorial Diagnostic Criteria [9] for the ALS-cohort, with and without accompanying frontotemporal dementia (FTD), respectively. Diagnoses were extracted from documentation of the DZNE registries. While data on disease severity and duration has been published for different patient cohorts of the DZNE registries elsewhere [10], no specific clinical information on symptoms or disease progression was obtained for the individual patients included in this morphometric study. We included only probands with adequate MRI imaging including artifact-free, thin sliced three-dimensional T1-weighted sequence at 3.0 T scanners, see below for details. Individuals with missing or inadequate MRI data were excluded. Patients without matching counterparts in the matching process were also generally excluded. Probands were matched for age (± 5 years of age) at the time point of their first study MRI, as well as sex. Healthy subjects from the DESCRIBE registries were added, also matched for age and sex. An exception had to be made for the ALS-FTD-group, because for six of the ten individuals included no matching partners in regards of age could be found, however, they were still matched regarding gender. 185 probands could be retrospectively included in the study. The collective included 36 cases of PSP, of which 14 were classified as PSP-FTD, 77 cases of ALS, of which 10 were classified as ALS-FTD, and 72 healthy controls (HC). All MRI datasets underwent random numerical pseudonymization before further evaluation.

### MRI Acquisition

All probands underwent standardised MRI protocols on 3.0 T MAGENTOM Verio MRI scanners (Siemens Healthineers AG, Forchheim, Germany), accredited for DZNE research, using a 32-channel head receiving coil. The protocols included a T1-weighted 3D magnetisation prepared rapid acquisition gradient echo (MPRAGE) sequence with 1 mm isotropic voxel size, which was used for planimetric and volumetric analysis.

### Morphometric Measurements

**Fig. 1:**
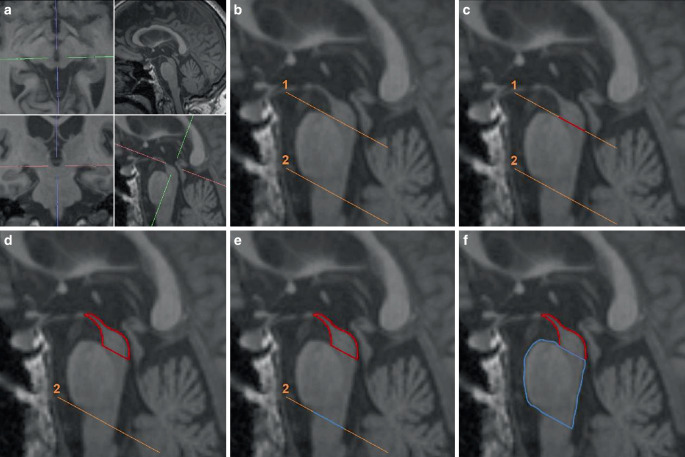
Workflow for manual planimetric measuring of the midsagittal midbrain and pons area as introduced by Oba et al. [11]. **a** The midsagittal plane was determined in multiplanar reformation as the sagittal plane containing the intersection between the mamillary bodies in the axial slice (upper left), cutting longitudinally through the aqueduct in the sagittal slice (lower right) and the anterior median fissure of the rostral medulla oblongata in the coronal view (lower left). The falx cerebri does not adequately define the midsagittal plane of the midbrain. **b** Two parallel auxiliary lines. The first (1) through the interpeduncular fossa at the ponto-mesencephal junction and the inferior edge of the quadrigeminal plate defines the caudal midbrain border. The second (2), drawn parallel to (1) through the pontomedullary junction, defines the caudal border of the pons. **c** and **d** The midsagittal midbrain area is measured starting with a straight line along (1) from the rostral “notch” to the aqueduct (sparing the aqueduct and the quadrigeminal plate) and then by continuously contouring the “hummingbird’s head” to the “tip of the beak” between the mamillary bodies, and back to the beginning at the mesencephalo-pontine “notch” (red line). **e** and **f** The midsagittal midbrain area is measured in an analogue way starting at the ponto-medullary junction, along line 2 to the fourth ventricle, and then continuously contouring the pons area (blue line)

The planimetric measurements of all 185 subjects were carried out by two investigators, blinded for diagnoses, with TeraRecon Aquarius Intuition Viewer Ver. 4.4.13.P2 (TeraRecon Inc., Durham, NC, USA). The MBA and the midsagittal pons area were manually measured in accordance with the literature [5, 11]. In the first step, the midsagittal plane was determined in multiplanar reconstructions of the 3D T1w MPRAGE sequence. In the resulting midsagittal plane, the midbrain area and the pons area were determined by manually drawing the defined contours. For an in-depth explanation of the manual planimetric measurements see Fig. 1. The resulting MBA and midsagittal pons areas were noted, and the MB/P-ratios were calculated. Forty datasets, including subjects from all groups, underwent re-pseudonymisation and were re-evaluated. Both investigators repeated manual morphometric measurements of MBA and midsagittal pons areas, blinded for the measurement results of the first round, in order to assess intra-rater reliability.

To ensure comparability of midbrain and pons sizes that may be affected by age, gender and head size, additional automated voxel-based morphometry (VBM) was performed with the Statistical Parametric Mapping software suite (SPM12, UCL Queen Square Institute of Neurology, London, UK). Using the standardised segmentation model of SPM12, the MRI scans were automatically segmented into grey matter (GM), white matter (WM), and cerebrospinal fluid (CSF) of the whole brain. No volumetric segmentation of different brain regions was carried out. The resulting “total intracranial volume (TIV)”, composed of GM, WM and CSF, and “brain volume (BV)”, composed of GM and WM, were used as covariates in the statistical analysis.

### Statistical Analysis

The statistical analysis was carried out with SPSS Version 27.0 (IBM Inc., NY, USA). P‑values of < 0.05 were regarded as statistically significant. Testing for group differences was done by chi-square test for the variable sex, and ANOVA for the variables age, total intracranial volume (TIV), and brain volume (BV). Differences of MBA and MB/P-ratios between the groups, corrected for age, sex, TIV and BV were analysed using univariate ANCOVA, followed by post-hoc-comparison with Bonferroni-correction. In addition, a receiver operating characteristic (ROC) analysis was performed to determine the quality of the morphometric imaging parameters as diagnostic tests in comparison to published data for atypical Parkinsonian syndromes. Inter-rater and intra-rater reliability were assessed by determining intraclass correlation coefficients.

## Results

An overview of demographic information and measurement results is given in Table 1. Of the 185 probands included in the study, the HC group consisted of 72 subjects with a median age of 69.49 ± 6.36 years, 32 (44.4%) of whom were female and 40 (55.6%) male. The group of PSP patients was considerably smaller, with 22 subjects with a mean age of 71.47 ± 6.10 years. Ten patients (45%) of this group were female, 12 male (55%). The ALS group consisted of 67 patients with a mean age of 68.89 ± 6.42 years, including 29 women (43.3%) and 38 men (56.7%). The ALS-FTD patients formed the smallest group with ten subjects with a mean age of 63.04 ± 10.63 years. The group included five women and five men (50%). The PSP-FTD group consisted of 14 patients with a mean age of 71.89 ± 7.25 years, including six women (43%) and eight men (57%). Statistical analysis revealed no significant differences in sex, TIV or BV between the groups. Regarding age, patients in the ALS-FTD-group were significantly younger compared to all other groups (ANOVA, *p* < 0.05 for all comparisons), owing to the inclusion of younger patients.

**Table 1:** Demographic information and morphometric measurement results

	HC	PSP	ALS	ALS-FTD	PSP-FTD
*n*	72	22	67	10	14
Age [years] (SD)	69.5 (6.36)	71.5 (6.10)	68.9 (6.42)	63.0 (10.63)^+^	71.9 (7.25)
Sex [male] (%)	40 (56%)	12 (55%)	38 (57%)	5 (50%)	8 (57%)
TIV [ml] (SD)	1435 (129.70)	1378 (138.23)	1445 (137.65)	1468 (237.65)	1443 (243.69)
BV [ml] (SD)	1037 (100.44)	922 (102.43)	1029 (96.10)	979 (188.80)	955 (169.32)
Midbrain area [mm^2^] (SD)	137.02 (20.40)	90.04 (14.47)*	140.31 (20.06)**	116.35 (32.26)***	104.33 (21.24)****
Pons area [mm^2^] (SD)	545.21 (59.08)	486.66 (59.47)^#^	547.78 (55.54)	522.68 (49.66)	515.79 (57.74)
MB/P-ratio (SD)	0.253 (0.036)	0.186 (0.031)^†^	0.258 (0.039) ^††^	0.223 (0.059) ^†††^	0.203 (0.042) ^††††^

*n* number, SD standard deviation, TIV total intracranial volume, BV brain volume

For further statistical analysis, Midbrain area, Pons area and MB/P-ratio were corrected for age, sex, total intracranial volume and brain volume. Levels of significance derived from pairwise post-hoc-comparison with Bonferroni-correction

^+^*p* < 0.05 vs. all other groups

^*^p < 0.001 vs. HC and vs. ALS

^**^p < 0.001 vs. ALS-FTD

^***^p < 0.001 vs. HC

^****^p < 0.001 vs. HC and vs. ALS (ALS vs. HC: *p* = 1.0; PSP-FTD vs. ALS-FTD: *p* = 1.0)

#*p* < 0.005 vs. HC and vs. ALS

†*p* < 0.001 vs. HC and vs. ALS

††*p* < 0.001 vs. ALS-FTD

†††*p* < 0.005 vs. HC

††††*p* < 0.001 vs. HC and vs. ALS

Midsagittal planimetric measurements showed strong inter-rater reliability, with intraclass correlation coefficients r = 0.980 (95%CI 0.973–0.985) for MBA and r = 0.951 (95%CI 0.903–0.972) for MB/P-ratio. Intra-rater reliabilities were also excellent, with intraclass correlation coefficients 0.984 for MBA (95% CI 0.966–0.992) and 0.980 for MB/P-ratio (95% CI 0.961–0.990) for rater A, and 0.982 for MBA (95% CI 0.966–0.990) and 0.964 for MB/P-ratio (95% CI 0.932–0.981) for rater B.

**Fig. 2:**
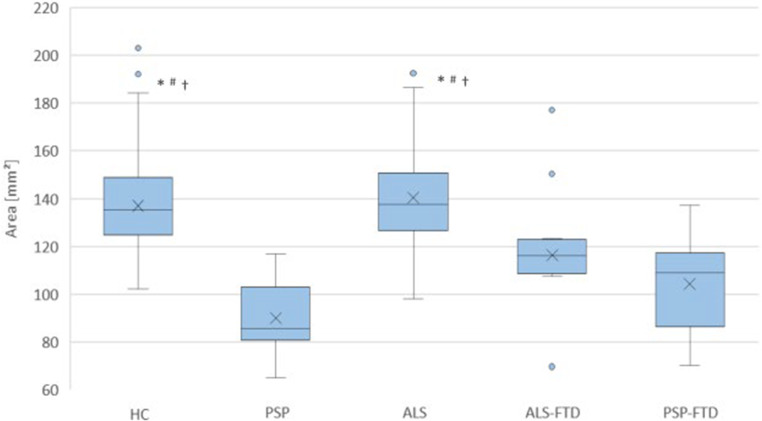
Comparison of midsagittal midbrain areas of healthy controls (HC, *n* = 72) and probands with classical progressive supranuclear palsy without FTD (PSP, *n* = 22), typical amyotrophic lateral sclerosis without FTD (ALS, *n* = 67), amyotrophic lateral sclerosis associated with frontotemporal dementia (ALS-FTD, *n* = 10) and progressive supranuclear palsy associated with frontotemporal dementia (PSP-FTD, *n* = 14). Dots: outliers > 1.5 interquartile range. ANCOVA, adjusting for sex, age, TIV and BV and post hoc comparison with Bonferroni-correction. (Asterisk) *p* < 0.001 vs. PSP, (Diamond) *p* < 0.001 vs. PSP-FTD, † *p* < 0.001 vs. ALS-FTD. No statistically significant differences between PSP, ALS-FTD and PSP-FTD, or between ALS and HC

The post-hoc comparisons revealed that the MBA and the MB/P-ratio were both significantly reduced in PSP with and without FTD when compared to HC and to ALS without FTD (Table 1). There were no statistically significant differences between ALS without FTD and HC. The MBA and the MB/P-ratio were significantly lower in both PSP-FTD and ALS-FTD when compared to HC. The MBA and the MB/P-ratio were significantly higher in ALS without FTD when compared to ALS-FTD. There were no statistically significant differences in any parameters between PSP-FTD and ALS-FTD. Figure 2 shows the exemplary results for the MBA.

**Fig. 3:**
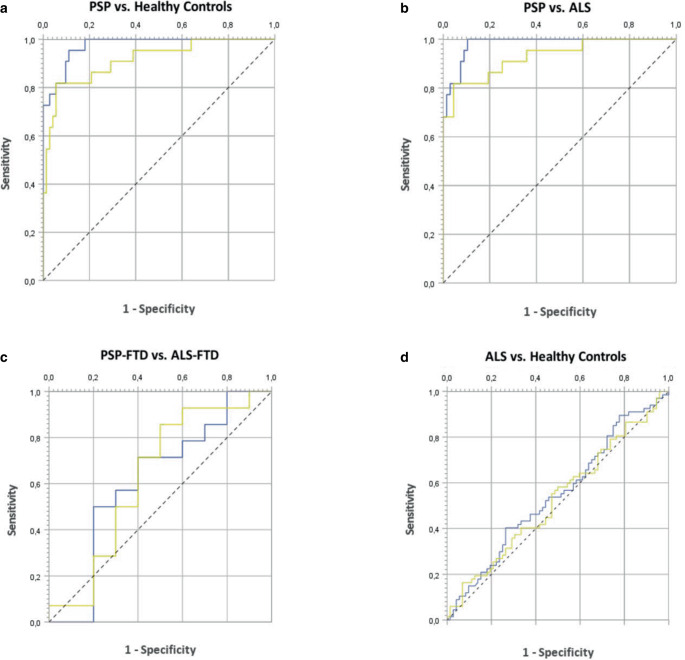
ROC-analysis of midsagittal midbrain area (MBA, blue) and midbrain-to-pons-ratio (MB/P-ratio, yellow) of select groups. **a** Excellent diagnostic accuracy of MBA to distinguish PSP without FTD from HC (AUC_MBA_ = 0.974, 95% CI 0.948–1.000) **b** Excellent diagnostic accuracy of MBA to distinguish PSP without FTD from ALS without FTD (AUC_MBA_ = 0.982, 95% CI 0.961–1.000). **c** Poor diagnostic accuracy of both MBA and MB/P for differentiating PSP-FTD from ALS-FTD (AUC_MBA_ = 0,614, 95% CI 0.366–0.862). **d** No parameter was able to differentiate between ALS and HC. Note: AUC = area under the ROC-curve. AUC results were defined as: 1.0 = perfect, > 0.8 = excellent, 0.7–0.8 good and < 0.7 poor performance

The ROC analyses showed excellent diagnostic accuracy regarding the MBA and the MB/P-ratio for discriminating PSP without FTD from HC with high area under the ROC-curve (AUC) values (AUC_MBA_ = 0.974, 95% CI 0.948–1.000; AUC_MB/__*P*_ = 0.916, 95% CI 0.845–0.987) and from ALS without FTD (AUC_MBA_ = 0.982, 95% CI 0.961–1.000; AUC_MB/__*P*_ = 0.930, 95% CI 0.864–0.996). However, they could not discriminate ALS without FTD from HC (AUC_MBA_ = 0.547, 95% CI 0.450–0.643; AUC_MB/__*P*_ = 0.528, 95% CI 0.432–0.625). Both parameters showed only poor diagnostic accuracy for the discrimination of ALS-FTD and PSP-FTD (AUC_MBA_ = 0.614, 95% CI 0.366–0.862; AUC_MB/__*P*_ = 0.629, 95% CI 0.382–0.875), as depicted in Fig. 3.

## Discussion

A clinical differentiation between ALS and PSP is challenging when both are associated with FTD and prominent upper motor neuron signs. No established biomarker is currently able to differentiate the two pathologies in these overlap syndromes (TDP-43 vs. Tau). Our goal was to assess whether the MBA and MB/P-ratio—as well-established imaging biomarkers in clinical neurodegenerative MRI assessments for Parkinsonian syndromes—might be applicable to the rare forms of ALS-FTD and PSP-FTD. We focused on these two imaging biomarkers using manual planimetric measurements because they are regularly used in our institution, may be performed easily and quickly in routine clinical MRI assessment and are known for excellent intra- and inter-rater reliability [5, 11], which was confirmed in our study. Our neuroimaging study shows that the midsagittal midbrain area and the midbrain-to-pons-ratio cannot differentiate between PSP and ALS with upper motor neuron signs when either are associated with FTD. The ROC analysis revealed insufficient diagnostic accuracy in this case. The curves of both the MBA and the MB/P-ratio were close to those of a random classifier. The area under the ROC curve (AUC) was substantially less than 0.7, which indicates poor performance. However, the MBA and the MB/P-ratio were able to distinguish between PSP and ALS to a high degree of accuracy when neither are associated with FTD. In these cases, the performance of both imaging parameters is comparable to data published by other groups regarding the differentiation between atypical Parkinsonian syndromes, as determined by ROC analysis, and we were able to reproduce the results for distinguishing PSP patients from healthy probands [5, 12]. This result can be explained by predominant whole midbrain atrophy being a hallmark of PSP-Richardson syndrome [13]. In contrast, midbrain atrophy is not dominant in ALS without FTD [6]. According to the literature, when midbrain and brain stem atrophy in ALS occurs, it is described to be most prominent in the pyramids of the medulla oblongata, in the pontine cranial nerve nuclei, and in the mesencephalic crura [14, 15]. As these structures lie outside of the midsagittal plane, it seems plausible that possible atrophy cannot be detected by MBA and MB/P-ratios as they are exclusively measured within the midsagittal plane. The whole brain volumetry analysis was here merely performed for statistical normalisation purposes, in order to adjust for potential differences related to the patients’ head size or brain volume. Therefore, we do not have data on regional atrophy patterns or midbrain and brain stem volume in our cases. Our study demonstrates that the midsagittal midbrain area is reduced in cases of ALS with FTD compared to HC, whereas in ALS without FTD it is not different from HC and significantly higher than in ALS-FTD. The midsagittal midbrain atrophy we detected in ALS-FTD is a feature typically found in PSP that we did not expect in the setting of a TPD-43 proteinopathy. This is, to our knowledge, a negative yet novel finding, not yet represented in the literature. A possible explanation lies in the combination of FTD pathology in addition to ALS pathology. However, there is ambiguity in the literature on the extent of midbrain atrophy in different FTD variants [16], that may mainly occur in late stages [17], and it generally remains unclear whether the midsagittal midbrain is affected. Recently, Cunningham et al. reported that MBA and MB/P-ratio were able to distinguish PSP from bvFTD with modest accuracy [18], performing substantially better in this scenario than in our ALS-FTD vs. PSP-FTD cases, which seems contradictory to the assumption that FTD may be the main cause of midsagittal midbrain atrophy in ALS-FTD. As we do not have histopathological confirmation of clinical diagnoses in our cohorts, no further insightful theory on the pathophysiological cause of midsagittal midbrain atrophy in ALS-FTD is evident. Although the ALS-FTD group was the smallest with only 10 subjects and had the most outliers (*n* = 3, albeit in both directions), we want to emphasize that the patients in this group were significantly younger and specifically not older and did not significantly differ in total intracranial volume or whole brain volume when compared to the other groups, hence age-related midbrain atrophy is unlikely. If no difference between ALS and ALS-FTD had been observed, we would have not been able to rule out a false negative because of age differences; on the contrary, the midbrain areas and midbrain-to-pons-ratios of HC and ALS patients were higher compared to ALS-FTD, despite being older, thus indicating that the results of reduced MBA and MB/P-ratio in ALS-FTD are robust. This specific finding may well explain why the midsagittal midbrain area and the midbrain-to-pons-ratio as morphometric parameters are unable to distinguish PSP-FTD from ALS-FTD.

In the future, more sophisticated morphometrical MRI imaging parameters would be beneficial for the investigation of these overlap syndromes to serve as a surrogate marker for differentiating Tau from TDP-43 pathology. Very recently, the prospective registry study DESCRIBE-ALS-FTD was initiated [7], promising a growing study population for future evaluation. In principle, Tau-PET [19] could provide another non-invasive diagnostic measure; however, compared to MRI it currently represents a scarce resource with significant limitations. Recently, convolutional neural network based automatic whole brain MRI volumetry and segmentation with distinct atrophy profile visualizations has become available [20, 21]. Granular assessments of atrophy distribution in the respective midbrain and brain-stem regions in addition to cortical regions typically affected in the different proteinopathies [22–24] would provide a promising way to verify midbrain atrophy in ALS-FTD and compare different atrophy patterns. At the same time, minimally invasive plasma biomarkers for both Tau and TPD-43 have been developed [25], which may revolutionise laboratory diagnostics in neurodegeneration and represent a promising possibility for verification of imaging biomarkers, compared to invasive brain biopsy or postmortem analysis.

### Limitations

This study is based on predetermined clinical diagnoses. Although the clinical diagnostic criteria for PSP and ALS are very specific, the present study does not provide definitive certainty regarding the underlying pathology, especially as results from postmortem histological analysis were not available. It was not possible to assess how far the subjects had progressed in their disease as no clinical information was available regarding the onset, duration and course of the disease, in addition to time of death of the individual patients. We acknowledge that statistical correction with the covariate “age” may only partly address this problem. Owing to the rarity of ALS-FTD cases in general, exceptions had to be made in the matching process regarding age for this group. In addition, there are inherent challenges of studying this particular cohort regarding compliance during scanner investigation. As a result, limitations resulting from the sample size are implicit.

## Conclusion

Although the midsagittal midbrain area and the midbrain-to-pons-ratio are morphometric parameters that have proven reliable in atypical parkinsonian syndromes, they cannot differentiate clinical overlap syndromes between PSP-FTD and ALS-FTD, which strengthens the necessity to establish novel imaging biomarkers as well as liquid biomarkers which are able to detect pathological Tau and TDP-43.
